# Some DNM2 mutations cause extremely severe congenital myopathy and phenocopy myotubular myopathy

**DOI:** 10.1186/s40478-018-0593-2

**Published:** 2018-09-12

**Authors:** Valérie Biancalana, Norma B. Romero, Inger Johanne Thuestad, Jaakko Ignatius, Janne Kataja, Maria Gardberg, Delphine Héron, Edoardo Malfatti, Anders Oldfors, Jocelyn Laporte

**Affiliations:** 1 Laboratoire Diagnostic Génétique, Faculté de Médecine, CHRU, Nouvel Hôpital Civil, 1 place de l’Hôpital, 67091 Strasbourg, France; 2 Institut de Génétique et de Biologie Moléculaire et Cellulaire, IGBMC, Illkirch, France; 3 Centre National de la Recherche Scientifique, CNRS UMR7104, Illkirch, France; 4 Institut National de la Santé et de la Recherche Médicale, INSERM, U964, Illkirch, France; 5 Université de Strasbourg, Illkirch, France; 6 Center for Research in Myology, GH Pitie-Salpêtrière, Sorbonne Universités, UPMC Univ Paris 06, INSERM UMRS974, CNRS FRE3617, Paris, France; 7 Neuromuscular Morphology Unit, Myology Institut, GH La Pitié-Salpêtrière, Paris, France; 8 Centre de référence de Pathologie Neuromusculaire Paris-Est, Institut de Myologie, CHU Paris-GH La Pitié-Salpêtrière, Assistance Publique-Hôpitaux de Paris, Paris, France; 9 Department of Pediatrics, Skane University Hospital, Malmo, Sweden; 10 Department of Clinical Genetics, Turku University Hospital, Turku, Finland; 11 Department of Paediatrics and Adolescent Medicine, Turku University Hospital, Turku, Finland; 12 Department of Pathology, Turku University Hospital and Institute of Biomedicine, Turku University, Turku, Finland; 13 Service de Génétique clinique et Médicale, CHU Paris-GH La Pitié-Salpêtrière, Assistance Publique-Hôpitaux de Paris, Paris, France; 14 Department of Pathology, and Genetics, University of Gothenburg, Sahlgrenska University Hospital, Gothenburg, Sweden

**Keywords:** *DNM2*, *MTM1*, Congenital myopathy, Centronuclear myopathy, Hypotonia

Centronuclear myopathies (CNM) are rare congenital myopathies characterized by muscle weakness with facial and eye involvement and intracellular disorganisation of myofibers with centralized nuclei [5, 8]. Several forms and mode of inheritance have been described. The most severe form, also called X-linked myotubular myopathy, is due to *MTM1* mutations and is associated with perinatal severe hypotonia and respiratory distress leading to death of most affected boys in infancy (MIM#310400) [7]. Dominant *DNM2* mutations are linked to milder cases with either neonatal, childhood or adult onset and proximal or diffuse muscle weakness (MIM#160150) [2, 3]. Previously described neonatal *DNM2* cases showed gradual improvement in motor function and survival into adulthood [1]. Unlike *MTM1*-CNM, reported cases of *DNM2*-CNM biopsies often show a radial distribution of sarcoplasmic strands on cross sections. Here we present three unrelated *DNM2*-CNM cases resembling myotubular myopathy at the clinical and histopathological levels.

Two girls and one boy from unrelated families presented at birth with global and severe hypotonia with respiratory distress requiring invasive and permanent respiratory support (Additional file 1: Table S1). Patients 1 and 2 had multiple contractures. Patient 1 is a male born at 29 weeks of estimated gestational age (EGA), presenting with foetal akinesia and disturbance of cardiac rhythm. Hydramnios was detected. He had a congenital and bilateral chylothorax and died at 5 weeks from a bronchopulmonary dysplasia. Patient 2 is a girl delivered at term by cesarean section due to monotonic heart rate. No amniotic fluid was present. She presented with small intracerebral hemorrhages but no major brain malformations at 1.5 months, and developed 40 degrees convex scoliosis by 4 months. Extubation attempt at 5 months failed and she died at 8 months of age from pneumonia. Patient 3 is a girl born at 34 weeks of EGA. She had a bilateral ptosis and high-arched palate. Brain MRI uncovered a leukoencephalopathy with enlarged ventricles and reduced white matter. She died at 4 months from respiratory failure.

Muscle biopsies were performed at 1 month from quadriceps for patients 1 and 3 and at autopsy at 8 months for patient 2. They showed fiber size variability and hypotrophic muscle fibers with prominent nuclear centralizations (Fig. 1a). No clusters of nuclei were observed (Additional file 1: Figure S1). NADH-TR staining revealed centrally located hyperintense reaction in the majority of fibers, without radial distribution of sarcoplasmic strands as the spokes of a wheel. Predominance of type 1 fiber was observed for patient 2 with more variability in fiber size and some increase in connective tissue. Electron microscopy ultrastructural analysis in patient 1 confirmed the presence of prominent nuclear centralizations. Of note centralized nuclei were surrounded by amorphous material and partially disorganized and misaligned sarcomeres. Satellite cells count appeared normal unlike neonates with myotubular myopathy in whom a decrease was noted [9](Fig. 1b).

**Fig. 1:**
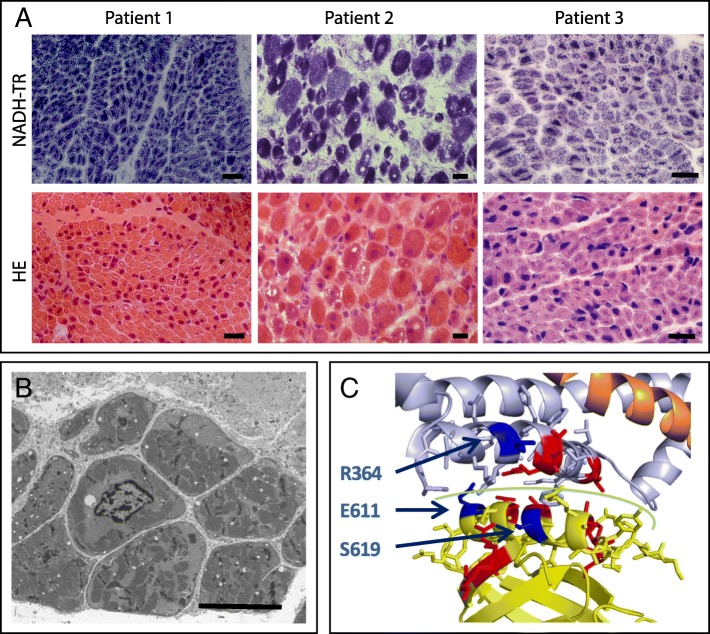
**a.** Hematoxilin-eosin (HE) and nicotinamide adenosine dinucleotide-tetrazolium reductase (NADH-TR) staining of muscles from the patients, showing fibers with centralized nuclei (HE) and abnormal central accumulation oxidative staining and a paler peripheral halo. Scale bars 20 μm. **b.** Electron microscopy of patient 1 muscle showing partial sarcomeres disorganisation and central nuclei. Scale bar 10 μm. **c.** Localization of presently reported mutations (dark blue) compared to known *DNM2*-CNM mutations (red) on the 3D model of nucleotide-free human DNM1 (PDB 3SNH). They all clusterize at the PH (yellow) – Middle/stalk (light blue) interface (green line)

*MTM1* mutations were excluded in patients 1 and 3. All the patients were found with heterozygous de novo *DNM2* mutations, NM_001005360.2: c.1831G > A - p.Glu611Lys, c.1090C > T - p.Arg364Cys, and c.1856C > T - p.Ser619Leu for patient 1, 2 and 3 respectively, through direct Sanger sequencing or an arthrogryposis gene panel (CeGaT, Tübingen, Germany). The p.Ser619Leu mutation was reported in at least 11 CNM cases with neonatal onset and a milder course compared to the present cohort. Mutations p.Glu611Lys and p.Arg364Cys are novel and are not found in gnomAD (http://gnomad.broadinstitute.org/). They affect aminoacids conserved down to drosophila and are predicted pathogenic by SIFT and Polyphen-2. Furthermore, they cluster with most known mutations on the 3D structure (Fig. 1c).

Here we report the most severe CNM patients with heterozygous *DNM2* mutations. Compared to previously reported *DNM2*-CNM cases [3], they were fully dependent on invasive ventilation and all died within the first months of life. The very early lethal outcome in patient 1 may have been influenced by concomitant prematurity. Nevertheless, the three patients did not improve except for a slight muscle strength enhancement appearing after 6 months of age in patient 2. Furthermore, early developmental milestones were delayed (Additional file 1: Table S1), in contrast with some previously described neonatal onset *DNM2* patients [4]. This study enlarges the clinical and genetic spectrum of *DNM2*-CNM. Moreover, it underlines that *DNM2* mutations can be associated with decreased survival.

In addition to a CNM phenotype, the 3 patients display similar features with the lethal congenital contracture syndrome (MIM#615368) due to a *DNM2* homozygous mutation [6], especially multiple contractures, fetal hypokinesia, pulmonary hypertension, brain hemorrhages, and abnormal fetal heart rythm.

The present *DNM2*-CNM cases were highly similar to myotubular myopathy due to *MTM1* mutations, although none of them presented with the association of facial hypotonia, ptosis, ophthalmoplegia and elongated face that is typical in *MTM1*-CNM cases. In addition to the perinatal severity, they had very severe hypotonia, respiratory distress and the same histopathological findings, lacking the radial strands hallmark of most other *DNM2* cases.

In conclusion, *DNM2* should be investigated in congenital myopathies presenting as myotubular myopathy.

## Additional file

Additional file 1: Clinical, molecular, histopathological and ultrastructural findings for the patients. **Table S1** Clinical and molecular findings in the *DNM2* severe cases. **Figure S1** Histopathological and ultrastructural findings for the patients. Patients 1, 2 and 3: Hematoxilin-eosin (HE) staining of muscles showing fibers with centralized nuclei. Patient 1: ATPase at pH 9.4 showing type I (pale) and type II (dark) fibers. Patient 3: ATPase at pH 4.6 showing type 1 fibers dark and type 2 fibers less stained. Scale bars 20 μm. (ZIP 46909 kb)

## Abbreviations

CNM: Centronuclear myopathies
DNM2: Dynamin 2
EGA: Estimated gestational age
HE: Hematoxilin-eosin
MRI: Magnetic resonance imaging
MTM1: Myotubularin 1
NADH-TR: Nicotinamide adenine dinucleotide tetrazolium reductase
