# Supplementary figures and images for: Some DNM2 mutations cause extremely severe congenital myopathy and phenocopy myotubular myopathy
Source: Acta Neuropathol Commun. 2018 Sep 12;6:93. doi: 10.1186/s40478-018-0593-2 (PMC6134552; doi:10.1186/s40478-018-0593-2)

Patient 1

Patient 2

Patient 3

HE

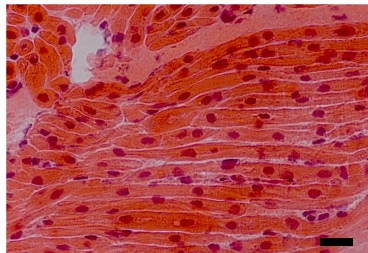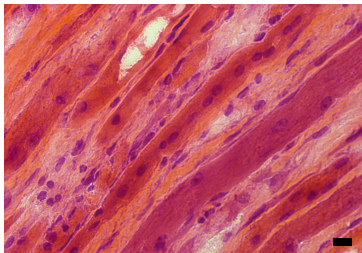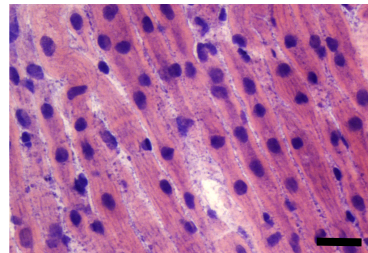

ATP

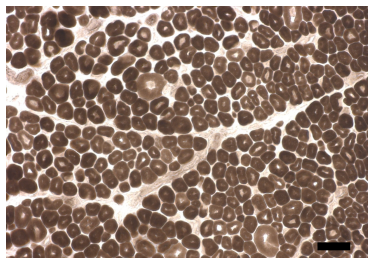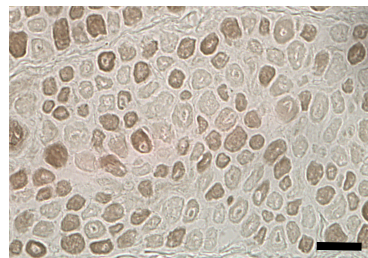

Supplement: Supplementary file 1 — Clinical, molecular, histopathological and ultrastructural findings for the patients. Table S1 Clinical and molecular findings in the DNM2 severe cases. Figure S1 Histopathological and ultrastructural findings for the patients. Patients 1, 2 and 3: Hematoxilin-eosin (HE) staining of muscles showing fibers with centralized nuclei. Patient 1: ATPase at pH 9.4 showing type I (pale) and type II (dark) fibers. Patient 3: ATPase at pH 4.6 showing type 1 fibers dark and type 2 fibers less stained. Scale bars 20 μm. (ZIP 46909 kb) [file 40478_2018_593_MOESM1_ESM.zip › Biancalana_DNM2_Figure S1.pdf]
